# Green Synthesis of Silver Nanoparticles and Evaluation of Their Antibacterial Activity against Multidrug-Resistant Bacteria and Wound Healing Efficacy Using a Murine Model

**DOI:** 10.3390/antibiotics9120902

**Published:** 2020-12-13

**Authors:** Vajravathi Lakkim, Madhava C. Reddy, Roja Rani Pallavali, Kakarla Raghava Reddy, Ch Venkata Reddy, Inamuddin, Anwar L. Bilgrami, Dakshayani Lomada

**Affiliations:** 1 Department of Genetics and Genomics, Yogi Vemana University, Kadapa, AP 516005, India; vajravathi@gmail.com; 2 Department of Biotechnology and Bioinformatics, Yogi Vemana University, Kadapa, AP 516005, India; cmreddy@yogivemanauniversity.ac.in (M.C.R.); p.rojarani.virology@gmail.com (R.R.P.); 3 School of Chemical and Biomolecular Engineering, The University of Sydney, Sydney, NSW 2006, Australia; raghava.kakarla@sydney.edu.au; 4 School of Mechanical Engineering, Yeungnam University, Gyeongsan 712-749, Korea; 5 Advanced Functional Materials Laboratory, Department of Applied Chemistry, Zakir Husain College of Engineering and Technology, Faculty of Engineering and Technology, Aligarh Muslim University, Aligarh, UP 202002, India; inamuddin@zhcet.ac.in; 6 Deanship of Scientific Research, King Abdulaziz University, Jeddah 80216, Saudi Arabia; alegman@kau.edu.sa

**Keywords:** silver nanoparticles, green synthesis method, *Catharanthus roseus*, *Azadirachta indica*, multidrug-resistant bacteria, wound healing

## Abstract

Green nanotechnology has significant applications in various biomedical science fields. In this study, green-synthesized silver nanoparticles, prepared by using *Catharanthus roseus* and *Azadirachta indica* extracts, were characterized using UV–Vis spectroscopy, dynamic light scattering, X-ray diffraction, scanning electron microscopy, and transmission electron microscopy. Silver nanoparticles (Ag NPs) synthesized from leaf extracts of *C. roseus* and *A. indica* effectively inhibited the growth of multidrug-resistant (MDR) bacteria isolated from patients with septic wound infections. The maximum bacteriolytic activity of the green-synthesized Ag NPs of *C. roseus* and *A. indica* against the MDR bacterium *K. Pneumoniae* was shown by a zone of inhibition of 19 and 16 mm, respectively. *C. roseus* Ag NPs exhibited more bacteriolytic activity than *A. indica* Ag NPs in terms of the zone of inhibition. Moreover, these particles were effective in healing wounds in BALB/c mice. Ag NPs of *C. roseus* and *A. indica* enhanced wound healing by 94% ± 1% and 87% ± 1%, respectively. Our data suggest that Ag NPs from *C. roseus* and *A. indicia* ameliorate excision wounds, and wound healing could be due to their effective antimicrobial activity against MDR bacteria. Hence, these Ag NPs could be potential therapeutic agents for the treatment of wounds.

## 1. Introduction

Burn injuries, wounds, and diabetic foot ulcers (DFUs) are global public health problems and a leading cause of mortality and amputations. The healing of burns, wounds, and DFUs involves a dynamic and complex network that requires continuous communication between cells in the form of cytokine release, cell-to-cell contacts, and cell-to-matrix interactions. The use of nanomedicines has increased enormously, and nanomaterials have been shown to offer promising strategies to optimize and improve the treatment of numerous disorders, including burns and wounds, owing to their unique small size, large surface area, and large surface-to-volume ratio. Hence, nanoparticles are considered magic bullets that are used in fundamental tasks in science, medicine, and different biotechnological fields, including imaging, biosensors, targeted drug delivery, and disease therapy [1,2]. More importantly, nanoparticles have played a role in delivering drugs, light, heat, and many substances to specific cancer cells in several biological applications. Metal nanoparticles are eminently illustrated as having antioxidant, anticancer, anti-inflammatory, and antimicrobial activities and play a role in wound healing [3,4,5]. Prior to antibiotic discovery, silver(Ag) was widely used as an antimicrobial agent to treat wound infections [6].However, after the discovery and abundant application of antibiotics, silver usage subsided because of its toxic nature and the easy applicability of antibiotics. Researchers have gained interest in using silver nanoparticles (Ag NPs) coupled with phytochemicals for use as antibacterial, antifungal, and anticancer agents [7].

Green synthesis of nanoparticles has gained attention because of its advantages, including being nontoxic, safe for humans, eco-friendly, and economically viable, compared to chemical and physical synthesis methods [8]. Instead of using silver alone, Ag NPs coupled with phytoextracts have gained more interest because of their action on bacterial and fungal pathogens and promotion of wound healing. Green-synthesized Ag NPs have been extensively used in biomedicine, purification of water, cosmetics, the food industry, numerous household products, and clothing [9,10]. Using plant extracts, Ag NPs synthesis has recently advanced, and it is now safe, allows convenient collection, and can utilize a wide range of metabolites for promoting the bioreduction of Ag^+^ (silver ions). Jha et al. demonstrated that plant leaf chemical constituents are precisely implicated in the lessening of silver ions and the formation of silver nanoparticles [11]. It has been shown that Ag NPs and crude phytoextracts successfully inhibit multidrug-resistant (MDR) bacterial growth [12,13,14,15]. The antifungal capacity of green-synthesized Ag NPs has gained only marginal attention from researchers in the field of plant pathology [2,16]. Biological methods implicated in Ag NPs synthesis utilize natural plant leaf extracts that act as both reductants and capping agents [17,18].

In folk medicine, *Catharanthus roseus* (*C. roseus*; also known as “Sadabahar” or “Madagascar periwinkle”), which belongs to the “Apocynaceae” family, is widely known as a significant medicinal plant that is used for the treatment of many maladies [19]. *C. roseus* leaves and roots are a source of fundamental anticancer drugs such as vincristine and vinblastine; its phytochemicals, including alkaloids, have been shown to have antihypertensive and anticancer effects [20]. Native to the Indian subcontinent, *Azadirachta indica* (*A. indica*) is another medicinal plant belonging to the “Meliaceae” family; it is typically grown in tropical and subtropical regions. *A. indica* is an abundant source of triterpenoid phytochemicals, like limonoids, that are enriched with powerful medicinal properties, including anti-inflammatory, antioxidant, anticancer, and anti-helminthic activities, as well as natural insecticidal activities [21]. *A. indica* leaves, cork, seeds, and oil are widely used in healthcare products and in Sidda, Unani, and Ayurveda medicine.

To control bacterial infections of septic wounds, many conventional antibiotics have been tested by researchers to maintain sterile conditions on wounds and possibly enhance their healing rate. However, due to the overconsumption of antibiotics, mutations in DNA, transposons, and R-plasmids of bacteria may result in the development of drug resistance [22]. Prolonged antibiotic administration may inhibit the growth of natural flora and also affect the synthesis of various biomolecules such as growth factors and cytokines. Worldwide, even in developed nations, skin infections due to microbes are the cause of approximately 42–65% of total skin-related morbidity, occurring particularly among children [23]. Ansari et al. demonstrated that microbial species such as *Staphylococcus aureus* were frequently colonized on human skin and wounds can cause several types of infections on the skin [24]. *S. aureus* infections on the skin’s soft tissue can spread intothe surroundings, causingsevere diseases such as bacteremia [25]. Silver nanoparticles promote the production of free oxygen radicals, which oxidize the bacterial molecular structure through the delivery of silver (Ag^+^) ions [26].

Biosynthesized Ag NPs have distinct advantages in medical fields, act as antimicrobials, and are used for drug delivery [27,28]. Because of their size advantage (7–20 nm), Ag NPs act as drug carriers that inhibit the growth of microbes and detoxify most microbial contaminations by disrupting cell membranes and blocking various biological molecules [29]. Biosynthesized Ag NPs are widely utilized as antibacterial components against several MDR bacteria and also reduce the development of antibiotic resistance. Because of their small size, biosynthesized Ag NPs accumulate on the cellular membranes of bacteria and cause an imbalance in microbial membrane integrity, leading to the death of the targeted bacteria, irrespective of their drug-resistant nature [30]. The mechanism of activity of biosynthesized Ag NPs is not completely understood; the significant general approaches of their activity are as follows:

- Biosynthesized Ag NPs release free silver ions that traverse into cells, causing further breakdown of adenosine triphosphate (ATP) generation and promotingthe replication of DNA.
- Ag NPs, along with Ag^+^ ions, enhance the production of reactive oxygen species (ROS) in an antioxidant mechanism.
- Ag NPs cause cell membrane damage directly.

Commonly described mechanisms begin with silver ion release [31], followed by ROS generation [13,32] and cell membrane damage, but many ambiguous findings have been reported. Generally, the metal form of silver (Ag) nanocrystallites represents an optical peak at 3KeV because of their surface plasmon resonance [33].

The current study is mainly focused on the green synthesis of Ag NPs from leaf extracts of *C. roseus* and *A. indica* and the characterization of their morphological and structural properties. Antibacterial efficacy of the characterized nanoparticles was evaluated using the isolates of septic wound infections, such as MDR *Escherichia coli*, *Klebsiella pneumoniae*, *Staphylococcus aureus*, and *Pseudomonas aeruginosa.* The wound-healing efficacy of Ag NPs of *C. roseus* and *A. indica* was determined by using in-vivo studies on BALB/c mice through wound excision models. Green-synthesized Ag NPs were produced without using any toxic chemicals or physical approaches and required a low concentration of leaf extract. The method we used to synthesize Ag NPs is easy, inexpensive, and simple to implement. Currently, there is an improved awareness of green synthesis as eco-friendly, stable, safe, and economical, and successful antimicrobial agents have a wide range of applications in healthcare products [34]. Phyto-derived Ag NPs are an especially important resource in the medical research field [35]. We synthesized Ag NPs from aqueous leaf extracts of *C. roseus* and *A. indica*; then, we characterized them via X-ray diffraction (XRD), Fourier-transform infrared spectroscopy (FT-IR), dynamic light scattering (DLS), scanning electron microscopy (SEM) with energy dispersive X-ray analysis (SEM-EDX), and transmission electron microscopy (TEM) analyses; finally, we showed their antibacterial efficacy against MDR bacteria and wound healing capacity using an animal model.

## 2. Results and Discussion

### 2.1. Bioreduction and Synthesis of Ag NPs

**Figure 1:**
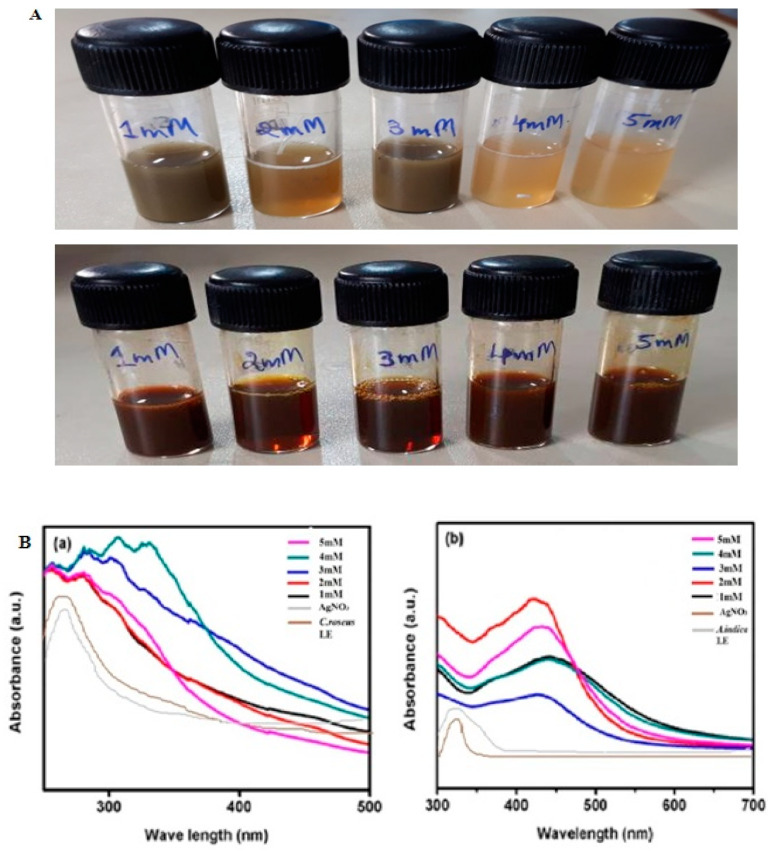
(**A**) Bioreduction of silver nanoparticles from *Catharanthus roseus* (top panel) and *Azadirachta indica* (bottom panel) leaf extracts. (**B**) UV–Vis spectroscopy analysis of biosynthesized silver nanoparticles of *Catharanthus roseus* (**a**) and *Azadirachta indica* (**b**) plant leaf extracts.

Ag NPs were synthesized from *C. roseus* (C Ag NPs) and *A. indica* (A Ag NPs) leaf extracts; the development of a brownish color was observed immediately after adding phytoextracts to 1 mM silver nitrate (AgNO_3_) due to the bioreduction of silver (Ag^+^) ions into silver nanoparticles (Ag NPs). Different concentrations (1to 5 mM) of AgNO_3_ with phytoextracts exhibited the appearance of a dark brown color, confirming the formation of Ag NPs, as shown in Figure 1A. The development of color was enhanced with time because of Ag^+^ reduction. During the synthesis of Ag NPs, due to the presence of biomass in the reaction mixture, the color changed from yellow-brown to darkish-brown. Here, biomass plays an essential role in the biosynthesis of Ag NPs. According to Mulvaney, the color change in the suspensions is due to the Ag NPs excitation of surface plasmonresonance vibrations [35]. Phytochemicals such as alkaloids, phenolic compounds, terpenoids, enzyme proteins, coenzymes, and sugars reduce metal (Ag) salts from a positive-oxidation state to a zero-oxidation state. The size and size distribution of metallic silver nanoparticles significantly depend on the biocompounds present in the extract. The presence of a strong reductantin the leaf extract enhances the bioreduction rate and favors the formation of smaller nanoparticles.

### 2.2. UV–Vis Spectra Analysis of Ag NPs

The structural characterization of green-synthesized nanoparticles was performed by UV–Vis spectral analysis. Mixing *C. roseus* and *A. indica* leaf extract suspensions with 1 to 5mM AgNO_3_ changed the color from light yellow-brown to dark brown, as mentioned above Figure 1A. As the reaction progressed, the color transformation revealed a reduction of Ag^+^ from silver nitrate to nanosilver, which was detected by the absorption maxima peak in the range of 300to 500 nm. The samples were observed periodically in the UV–Vis spectrometer at various concentrations of AgNO_3_ (1 to 5 mM), along with AgNO_3_ alone and phytoextracts without the addition of AgNO_3_. At the same time, the samples were monitored by UV–Vis spectroscopy, which revealed a sharp peak at 315–360 nm in *C. roseus* and at 410–440 nm in *A. indica*, as shown in Figure 1B. These data confirmed the formation of Ag NPs from the phytoextracts. We did not observe an increase in absorbance with increasing concentrations of AgNO_3_ with C Ag NPs. The lack of dependence on increasing concentration in C Ag NPs may be due to their particle size formation during the synthesis of Ag NPs from *C. roseus* phytoextract with AgNO_3._ This may be because an increase in concentration increases the density of nanoparticles. Furthermore, the surface plasmonresonance (SPR) peak gradually shifted towards red with respect to the concentration. The shift towards red indicates that the particle size gradually increases with concentration. Curve sharpness also increased with concentration, which may be due to the formation of spherical and cubical nanoparticles. This is illustrated in the UV–Vis spectra through the rise in absorbance and shift of the SPR peaks [36,37,38]. In biologically synthesized nanoparticles, there was a considerable shift in the absorption maxima. Narayan et al. showed that the free electrons in metal nanoparticles allowed the SPR absorption band in the UV–Vis spectrum [7]. Such a characteristic change in color was due to the excitation of SPR in the metal nanoparticles of the plant leaf extracts that reacted with the 1 mM silver nitrate (AgNO_3_) suspension. The UV–Vis spectra of the reaction mixture, at a range of wavelengths varying within 300–600 nm, showed a sharp peak at 320–335 and 420–440 nm in *C. roseus* and *A. indica*, respectively [36,37]. According to Udayasoorian et al., a sharp absorption band illustrates a spherical shape, and two other SPR bands are related to anisotropic particles [38]. According to Wiley et al., this method was used to investigate the shape and size of nanoparticles in liquid solutions [39].Hence, the UV–Vis spectroscopic findings verified that *C. roseus* and *A. indica* phytoextracts reduce silver-to-silver nanoparticles (Ag NPs). Bhakya and coworkers observed that peaks exploit the cubic structure and crystalline properties of biosynthesized silver nanoparticles in nanoscale units [40].

### 2.3. X-ray Diffraction Studies

**Figure 2:**
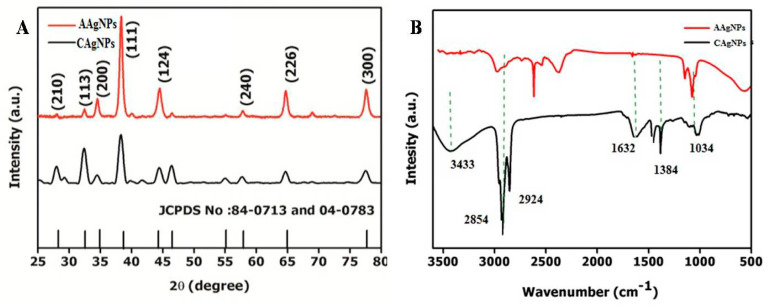
(**A**) X-ray diffraction (XRD) analysis of *Catharanthus roseus* silver nanoparticles (C Ag NPs) and *Azadirachta indica* silver nanoparticles (A Ag NPs).(**B**) FTIR spectra of *Catharanthus roseus* silver nanoparticles (C Ag NPs) and *Azadirachta* *indica* silver nanoparticles (A Ag NPs).

The crystalline nature of the green-synthesized Ag NPs from plant leaf extracts demonstrated that specific peaks were observed in the spectra analysis using the X-ray diffraction (XRD) method (Rigaku, Miniflex). The Ag NPs X-ray diffraction spectrum demonstrated sharp scattering peaks at 2θ = 27.9°, 32.2°, 34.6°, 38.4°, 44.2°, 57.8°, 64.7°, and 77.4°, corresponding to the (210), (113), (200), (111), (124), (240), (226), and (300) planes of the face-centered cubic crystal structure for silver (Ag), as depicted in Figure 2A. Four Bragg’s reflection patterns at 2θ = 32.2°, 38.4°, 44.2°, and 64.7° and in the spectrum varying in the range of 10–90 are represented by the conventional XRD method. The XRD spectrum patterns were considerably associated with (113), (111), (124), and (240). The lattice planes in the XRD spectrum were confirmed and crosschecked with the standard referral peak values (JCPDS Files no. 84-0173 and 04-0783), demonstrating that the synthesized Ag NPs were crystalline. The XRD spectra showed that the *C. roseus* and *A. indica* leaf extracts produced Ag NPs, and their crystalline nature was confirmed through the XRD method. The XRD peaks of the green-synthesized Ag NPs, with reference values, showed that Ag NPs were produced. The formed nanoparticles were confirmed via sharp peaks, and their 2θ values were 27.9°, 32.2°, 34.6°, 38.4°, 44.2°, 57.8°, 64.7°, and 77.4°, corresponding to (210), (113), (200), (111), (124), (240), (226), and (300), respectively, for Ag. Our XRD results demonstrated that the Ag NPs produced through the reduction of silver (Ag^+^) ions with *C. roseus* and *A. indica* extracts were crystalline in nature. Our data are consistent with earlier reports [41,42]. Unidentified peaks (peaks 27.9°, 34.6°, and 57.9°) were observed, and it was recognized that the phytochemicals acted as capping agents for Ag NPs production [43]. In addition, Ag NPs (around 20 nm) with a face-centered crystalline cubic structure were confirmed. Shameli et al. demonstrated that the XRD pattern of Ag NPs showed a crystalline property in cubic form [44]. Bhakya and coworkers observed that peaks indicate a cubic structure and the crystalline properties of biosynthesized Ag NPs in nanoscale units [40].

### 2.4. FTIR Analysis

A conventional Fourier transform infrared (FTIR) approach was used to identify secondary metabolites involved in reducing and capping for the synthesis of Ag NPs. FTIR spectra were recorded by employing potassium bromide (KBr) disks using an FTIR spectrometer (Perkin Elmer, Spectrum 2) with a wavenumber of 4000 to 400 cm^−1^. The characteristic FTIR spectrum of green-synthesized Ag NPs, as depicted in Figure 2B, showed peaks at 3433, 2854, 2924, 1632, 1384, and 1034 cm^−1^. In this spectrum, a sharp absorption signal peak at 3433 cm^−1^ is related to the N–H bond of amine groups of green-synthesized Ag NPs and provides stabilization of Ag NPs. Hence, the occurrence of N–H group-specific proteins and enzymes correspond to the reduction of AgNO_3_ to Ag [32]. A comparative study of the FTIR spectrum ofthe control shows the most important signal peaks of ~3433, 1632, and 1384 cm^−1^, illustrating the N–H group’s presence on the surface of Ag NPs. According to Mishra et al., the cell-free extract might contain biomolecules such as peptides and proteins that affect the formation of Ag NPs through reduction [45].

**Figure A1:**
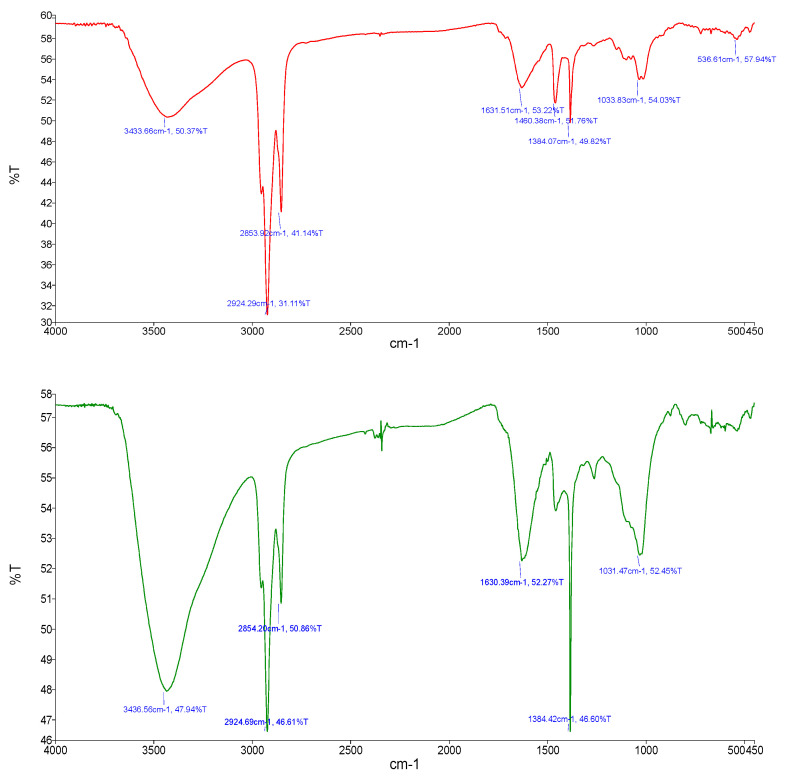
FTIR spectra of *C. roseus* (upper panel) and *A. indica* (lower panel) silver nanoparticles.

The wide peak at 3433 cm^−1^ demonstratesan amide (N–H) stretching vibration of the NH_2_ group, and OH with overlapping stretching vibration for water is recognized in *A. indica*. FTIR visible peaks with N–H and OH bond stretching at 3433 cm^−1^ clearly illustratesthe functional groups present in the green-synthesized *C. roseus* and *A. indica* Ag NPs, as shown individually in Appendix A and Figure A1. Another peak at 1632 cm^−1^ belongs to the stretching of C=O and a sharp signal peak at 2854 cm^−1^ could berelatedto the alkyne group in the leaf extracts of *C. roseus* and *A. indica*. The visible sharp signals at 1384 cm^−1^ represent C–O–C and C–O bonds. These visible signals are mainly related to flavonoids and terpenoids specifically present in plant extracts of *C. roseus* and *A. indica* [46,47]. These findings are in agreement with existing literature and confirmed that many bioorganic constituents from *C. roseus* and *A. indica* extracts produced persistent capping agents on green-synthesized Ag NPs [48].

### 2.5. Dynamic Light Scattering

**Figure 3:**
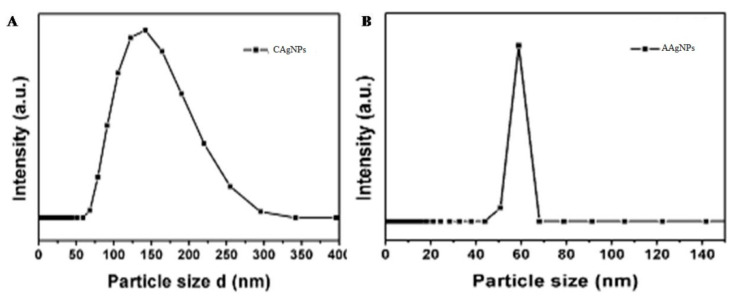
The hydrodynamic size determination of green-synthesized Ag NPs of *Catharanthus roseus* (**A**) and *Azadirachta indica* (**B**) by dynamic light scattering (DLS).

**Figure A2:**
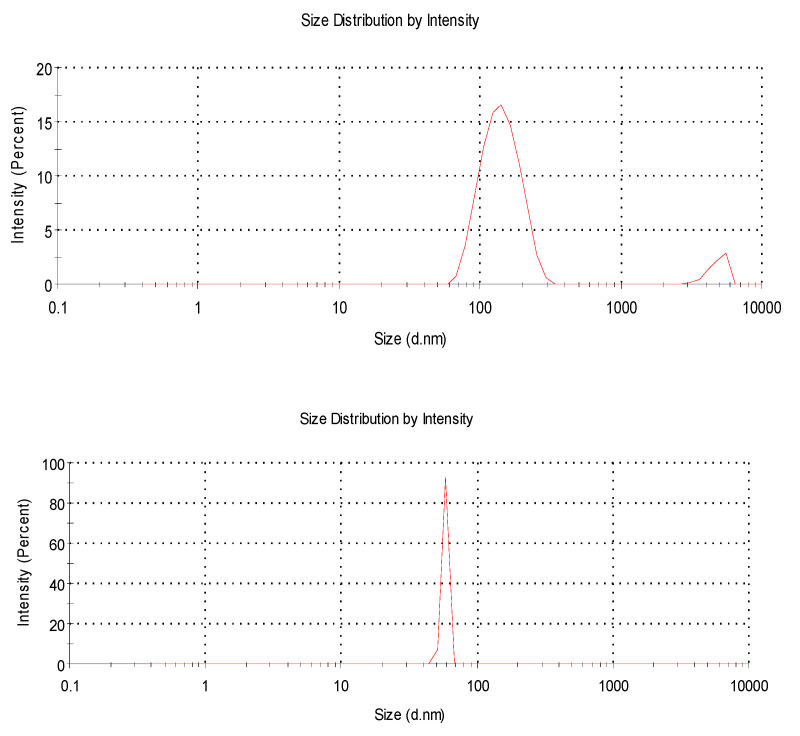
Hydrodynamic size determination of green-synthesized Ag NPs of *C. roseus* (upper panel) and *A. indica* (lower panel), as determined by dynamic light scattering (DLS).

We measured the distribution, typical particle size, and polydispersity index (PDI) of green-synthesized Ag NPs using dynamic light scattering (DLS).DLS data illustrated that the Ag NPs produced by the green route had a 31.4 diameter with Z, as reported by the distributions of size in number percentage. Briefly, 20.5 nm is related to q^O^ (number density distribution) and is based on the number percentage correlated with a polydispersity index of 0.65, demonstrating that the green-synthesized Ag NPs were greatly diffusive in suspensions (Figure 3). Using this approach, it was found that the nanoparticles hydrodynamic diameter was greater than the original diameter obtained from SEM and TEM images. Hence, due to electrostatic repulsions of the green-synthesized Ag NPs, the zeta potential value −15.2 represents great stability in water. The bioorganic constituents in the suspension serve as spacers to avoid agglomeration between Ag NPs. The TEM images assist the steric stabilization process, and the DLS study showed that Ag NPs biosynthesized through the green route resulted in a 35.69 nm average diameter. A polydispersity index (PDI) of 0.56 demonstrated that the green-synthesized Ag NPs were widely dispersed in a liquid medium. In DLS, the average particle size of C Ag NPs was 110 nm, with a range of 80–250 nm, and the average particle size of A Ag NPs was 60 nm, with various ranges of 40–80 nm, as indicated in Figure A2.

### 2.6. Scanning Electron Microscopy

The morphology of the green-synthesized Ag NPs was analyzed by scanning electron microscopy [49], and the pictures showthe cubic structure and unique shape of the nanoparticles produced with a 48–67 nm diameter range.

Analysis by the energy-dispersive X-ray (EDX) spectrum revealed that the incidence of silver elemental signals confirmed that the formed particles were Ag NPs through the bioreduction mechanism of leaf extracts with AgNO_3_. The *y*-axis represents the number of X-ray counts, and the *x*-axis represents energy in keV. The sharp elemental signal peaks showed the most significant emission energies for Ag, and these lines in the EDX spectrum confirmed that Ag had been properly recognized.

**Figure 4:**
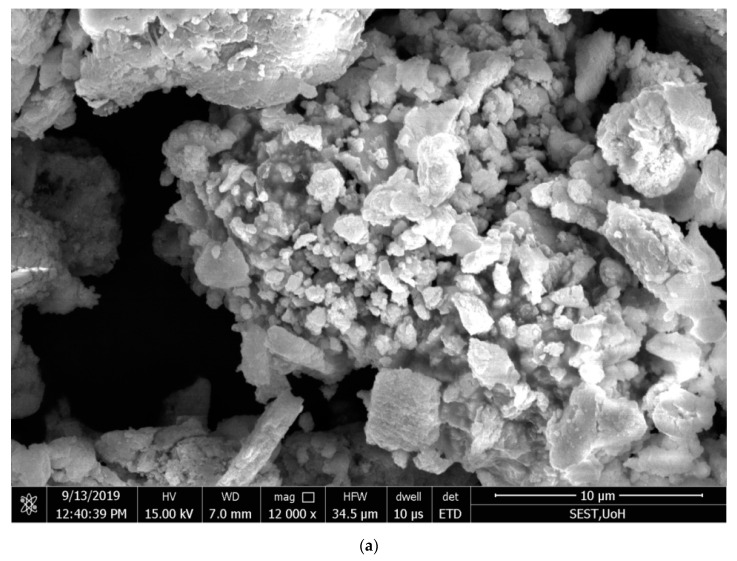

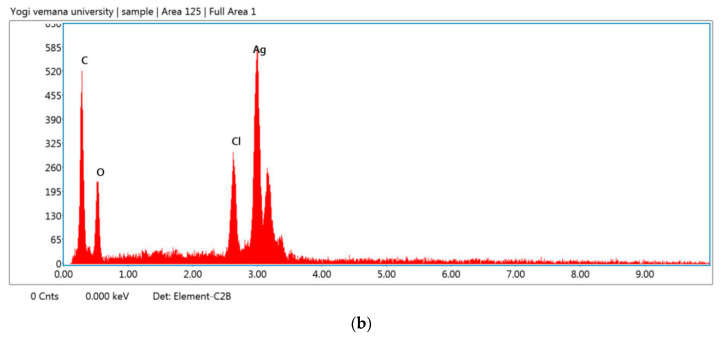
(**a**) Scanning electron microscopy(SEM) images of synthesized Ag NPs from *Catharanthus roseus*; (**b**) energy dispersive X-ray (EDX) spectrum of C Ag NPs showing the presence of different phytoelements as capping agents.

**Figure 5:**
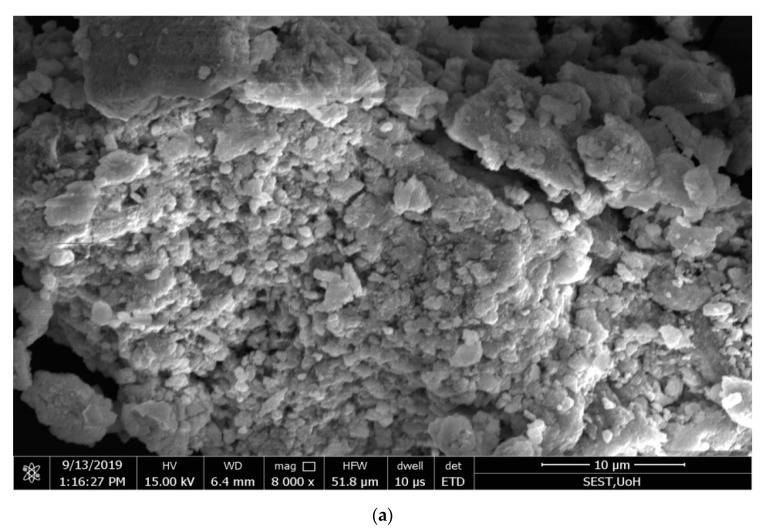

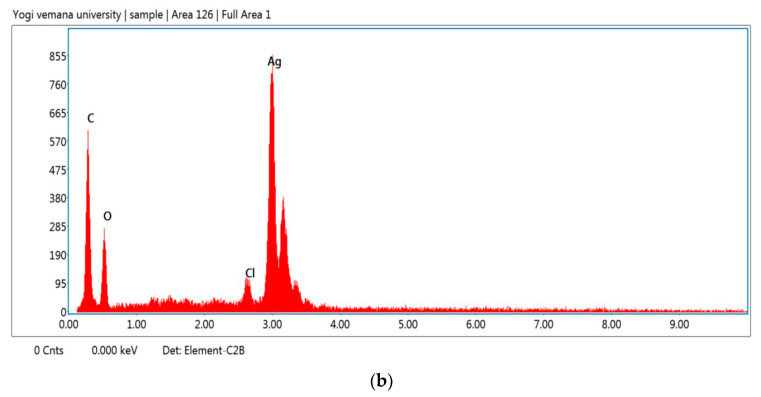
(**a**) Scanning electron microscopy (SEM) images of synthesized Ag NPs from *Azadirachta indica*; (**b**) energy dispersive X-ray (EDX) spectrum of A Ag NPs showing the presence of different phytoelements as capping agents.

The silver peak was from Ag NPs, and its atomic percentage was 13.1% in *C. roseus* and 19.9% in *A. indica*, as shown in Figure 4 and Figure 5, respectively. In *C. roseus*, except for Ag, the atomic percentages of carbon (C), chlorine (Cl), oxygen (O), and aluminum (Al) were 48.5%, 30%, 6.7%, and 0.9%, respectively. In *A. indica*, except for Ag, the atomic percentages of carbon (C), chlorine (Cl), oxygen (O), and aluminum (Al) were 38.5%, 38.5%, 1.7%, and 1.2%, respectively.The peak of carbon in the spectrum represents the adsorbed constituents of the leaf extracts, and the other peaks of oxygen and chlorine are formed because of plant element adsorption over Ag NPs. Carbon is a fundamental chemical constituent in both the *C. roseus* and *A. indica* leaf chemical structures. In *C. roseus* and *A. indica*, carbon sources are abundant in leaf extracts. The synthesized Ag NPs were washed several times with double-distilled water after synthesis to minimize contamination. Because ofthe agglomeration of phytochemicals present on the Ag NPs, there was a high amount of carbon on the green-synthesized Ag NPs. The presence of a high content of aluminum (Al) was probably due to the inclusion of the microscope stage in the analysis. Apart from carbon, the remaining elements showed a drastically decreased atomic percentage compared with silver; the EDX spectrum gives evidence of formed particles through bioreduction of plant leaf extracts, with AgNO_3_ confirmed as Ag NPs.

### 2.7. Transmission Electron Microscopy

**Figure 6:**
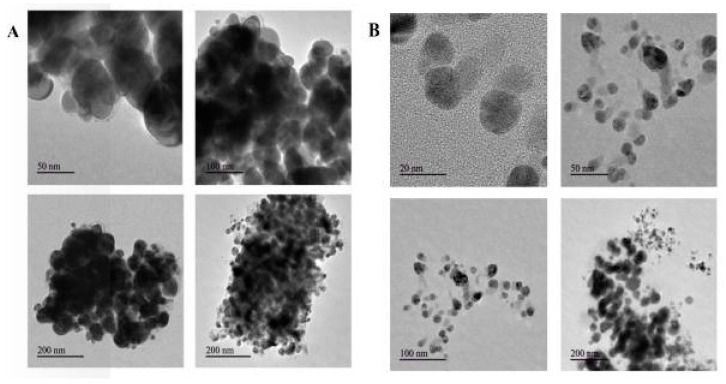
Transmission electron microscopy (TEM) images of biosynthesized silver nanoparticles from (**A**) *Catharanthus roseus* and (**B**) *Azadirachta indica* plant extracts.

The morphology (size and shape) of C Ag NPs and A Ag NPs were analyzed using TEM (JEOL, Japan) at an operating voltage of 200 kV. The synthesized C Ag NPs and A Ag NPs were transferred into a new vial. The sample was prepared by mixing with 95% alcohol and then 15 min of ultra-sonication in an ultrasonic water bath. A nanoparticlesaqueous solution (5 µL) was placed on coated carbon grids and air-dried immediately before screening. TEM grids were prepared by placing a drop of particle solution on a carbon-coated Cu grid and drying under a lamp. The characteristics of the Ag NPs synthesized using *C. roseus* and *A. indica* leaf aqueous extracts were examined using TEM. Ag NPs acquired through the green route with 10% (*w*/*v*) of *C. roseus* and *A. indica* leaf extracts in 1 mM AgNO_3_ showed particle sizes ranging 10–200 nm (average diameter 30 nm), as shown in Figure 6.

TEM images of the green-synthesized Ag NPs revealed that silver nanoparticles were predominantly spherical in shape; a few agglomerated Ag NPs were also observed, which indicates possible sedimentation at a later time. *C. roseus* TEM images revealed that there was variation in particle sizes, with a range from 20 to 50 nm, and the average particle size was found to be 30 nm [50]. The TEM images showed that the Ag NPs were agglomerated and embedded in a dense, thick pattern, possibly acting as stabilizing chemical constituents in the leaf extracts of *C. roseus* and *A. indica*.

### 2.8. Antibacterial Activity of Silver Nanoparticles

#### 2.8.1. Antibiotic Susceptibility Test

**Figure 7:**
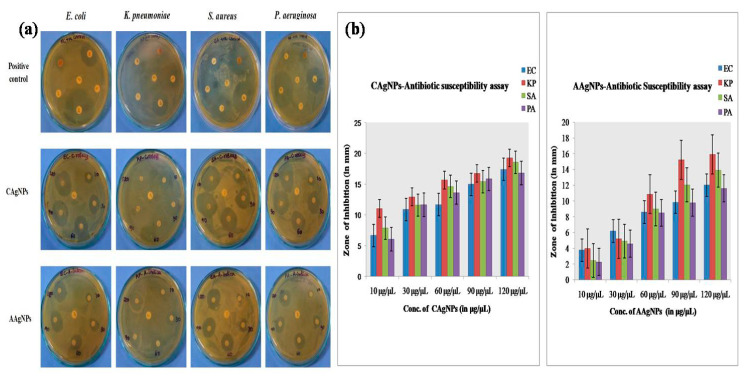
(**a**)Antibiotic susceptibility activity of biosynthesized silver nanoparticles (C Ag NPs and A Ag NPs) demonstrated by the disc diffusion method in multidrug-resistant (MDR) bacteria. (**b**) Graphical representation of antibiotic susceptibility activity of green-synthesized Ag NPs. EC—*Escherichia coli*, KP—*Klebsiella pneumoniae*, SA—*Staphylococcus aureus*, and PA—*Pseudomonas aeruginosa*.

The green-synthesized C Ag NPs and A Ag NPs showed effective bacteriolytic activity against MDR bacteria (*E. coli*, *K. pneumoniae*, *S. aureus*, and *P. aeruginosa*) isolated from wound infections. The green-synthesized C Ag NPs showed high antibacterial activity against both gram-negative (*E. coli*, *K. pneumonia*, *P. aeruginosa*) and gram-positive (*S. aureus)* bacteriaby showing a wider range of inhibitory zones compared to A Ag NPs at various concentrations (10, 30, 60, 90, and 120 µg/µL), as shown in Figure 7 and Table 1. The maximum bacteriolytic activity of green-synthesized C Ag NPs and A Ag NPs was shown as 19and 16-mm zones of inhibition at the highest concentration against *K. pneumoniae*, respectively. C Ag NPs showed more bacteriolytic activity at all tested concentrations compared to A Ag NPs, as described in Table 1.The saturation dose of Ag NPs for that specific tested species was optimized at lower concentrations, so the inhibition zone did not increase even when the doses of Ag NPs were increased. The exact mechanism of green-synthesized Ag NPs dose-exclusion in MDR bacteria is still unknown.

#### 2.8.2. Bacterial Reduction Assay

**Figure 8:**
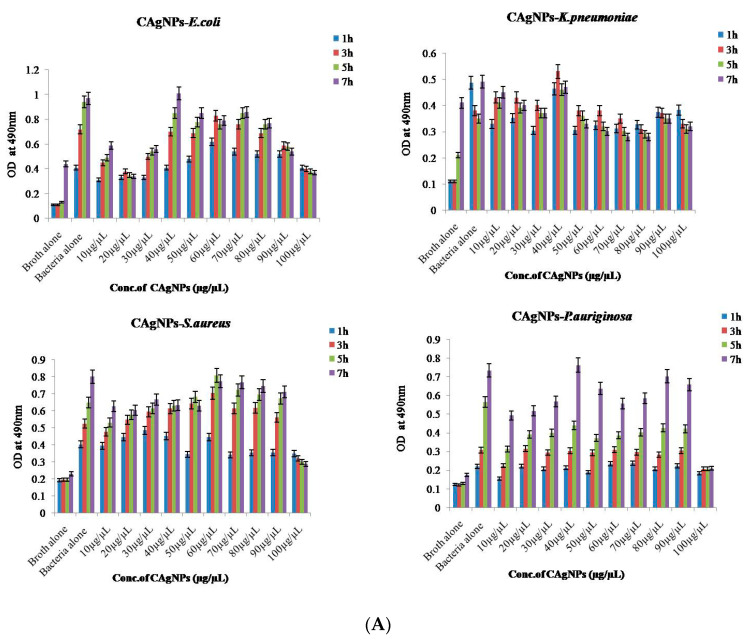

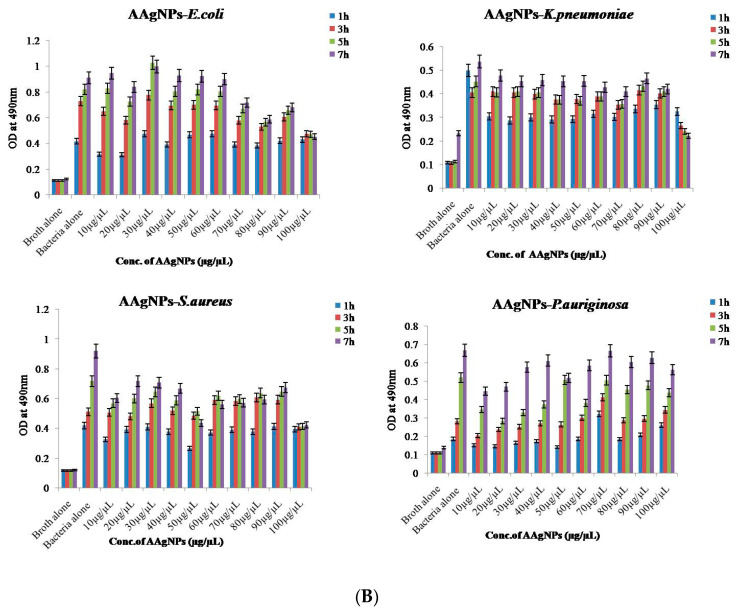
(**A**) Turbidity test for *C. roseus* Ag NPs (C Ag NPs) in MDR bacteria. (**B**) Turbidity test for *A. indica* Ag NPs (A Ag NPs) in MDR bacteria.

The bacterial growth inhibitory activity of C Ag NPs and A Ag NPs against MDR bacteria (*E. coli*, *K. pneumoniae*, *S. aureus*,and *P. aeruginosa*) at various time points (1, 3, 5, and 7 h of incubation) and concentrations (ranging from 10 to 100 µg/µL) were measured using a bacterial reduction assay. Interestingly, *P. aeruginosa* (80 µg/µL) required a higher amount of green-synthesized nanoparticles of *C. roseus* and *A. indica* to inhibit growth than the gram-positive bacteria *S. aureus* (10 µg/µL). For *K. pneumoniae* and *E. coli*, 10 µg/µL of synthesized nanoparticles was required for the inhibition of growth of MDR bacteria. The nanoparticles that were green-synthesized with different concentrations of *A. indica* and *C. roseus* were proven effective antibacterial agents against MDR bacteria. The results for C Ag NPs and A Ag NPs are represented in Figure 8A,B, respectively. The bacterial growth on MH-Agar (Mueller-Hinton agar) of *E. coli* and *K. pneumoniae* showed that lower concentrations of C Ag NPs (40–50 µg/µL) are optimum for inhibition. There is no significant inhibitory action obtained by increasing the concentration of C Ag NPs on MH-Agar for *E. coli* and *K. pneumoniae*. Therefore, 50 µL is the optimum/saturated concentration required for inhibitory action on *S. aureus* and *P. Aeruginosa* by C Ag NPs. No significant inhibitory action on bacterial growth was observedby increasing the dose volume of C Ag NPs. The tested species, especially *S. aureus* and *P. aeruginosa*, probably follow the dose-exclusion mechanism.However, at the highest concentration (100 µL), we found bacteriolytic activity in almost all strains. The exact mechanism of the inhibitory action of green-synthesized Ag NPs on bacterial growth is not known. The green-synthesized C Ag NPs and A Ag NPs showed significant bacteriolytic activity compared with several other plant extracts alone or silver nitrate alone. Yliniemi et al. demonstrated that the biosynthesized Ag NPs effectively cause cell death of MDR bacteria through various mechanisms of action on bacterial respiration and cell membrane permeability [51]. Rai et al. and other groups revealed that the smaller size of biosynthesized Ag NPs provides a large surface area, which ensures a more significant outcome compared with commercial silver (Ag) on bacteria [52,53,54]. Gurunathan et al. demonstrated the dose-dependent bactericidal activity of Ag NPs at concentrations ranging from 0.1 to 1.0 μg mL^−1^ against two gram-negative and two gram-positive bacterial strains. They showed that the antibacterial activity of Ag NPs at low concentrations was more effective against gram-negative bacteria than gram-positive bacteria. They found that cell viability was reduced, and no growth at MIC values was observed for both strains. Thus, the bactericidal effect depends on the concentration, and it is specific for each bacterial strain. Positively charged Ag NPs show bactericidal and bacteriostatic activity, as reviewed by Roy et al. (2019). They found that the antibacterial activity of Ag NPs at a 100 µg mL^−1^ concentration was slightly higher than that at 450 µg mL^−1^ compared to the control [55,56]. Our data demonstrated that although there was no dependence on concentration, at a higher concentration (100 µL), we observed bactericidal activity using a bacterial reduction assay.

According to Sahayaraj et al., biosynthesized Ag NPs further attached to the cell surface of bacteria and entered the bacteria, leading to DNA replication, interruption of adenosine triphosphate (ATP) production, and ROS generation, directly affecting the cell framework [57]. Moreover, the silver bactericidal effect was possibly related to the inactivation of phosphomannose isomerase catalysis and is involved in the transition of mannose-6-phosphate to fructose -6-phosphate, a key arbitrate in glycolysis and a common sugar catabolism mechanism in microorganisms [58].

### 2.9. Wound-Healing Efficacy of Silver Nanoparticles In Vivo

**Figure 9:**
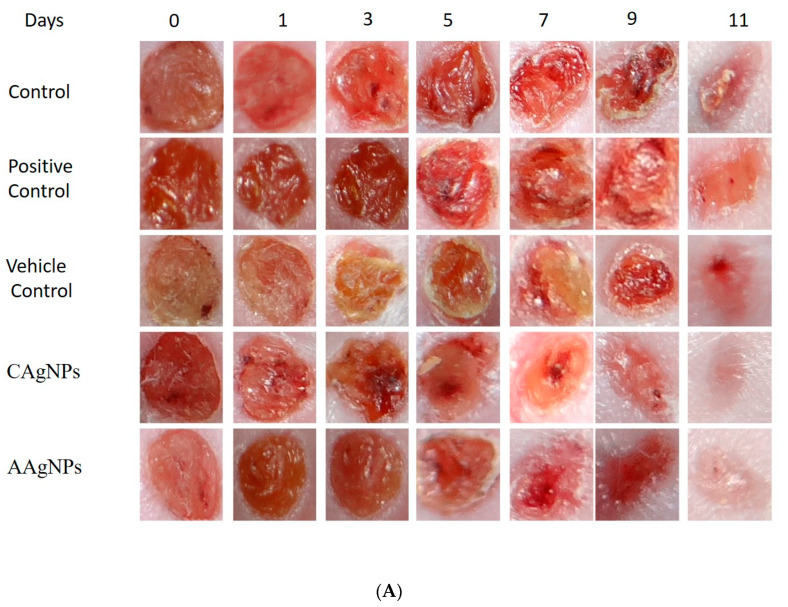

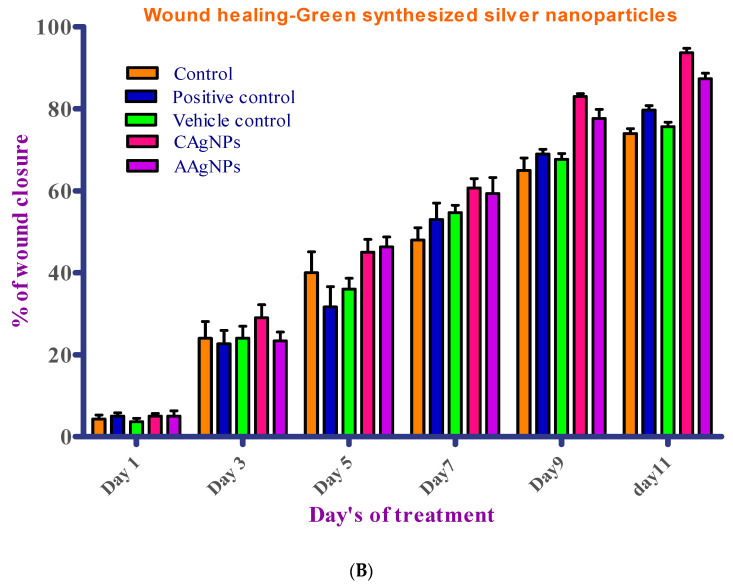
(**A**) Representative photographs showing wound closure after treatment with green-synthesized silver nanoparticles of *C. roseus* and *A. indica* (1% *w*/*w*) and control groups on days 0, 1, 3, 5, 7, 9, and 11. (**B**) Effect of topical application of green-synthesized silver nanoparticles of *C. roseus* and *A. indica* (1% *w*/*w*) on percent wound closure on different days in BALB/c mice.

The wound-healing efficacy of C Ag NPs and A Ag NPs was evaluated using female BALB/c mice using an excision wound-healing model with 5 mm biopsy punches. The wounds were generated on the skin surface dorsally, nano-formulations were applied on alternate days (on days 1, 3, 5, 7, 9, 11), and pictures were taken. Povidone–iodine ointment that is available on the market was used as a positive control for treated mice. As depicted in Figure 9A, green-synthesized silver nanoparticles (C Ag NPs and A Ag NPs)-treated mice showed enhanced wound constriction efficacy when compared to control and positive-control groups. The wound beds where the green-synthesized Ag NPs were topically applied showed no microbial growth, hemorrhage, or formation of pus throughout treatment, while the control group wounds showed remarkable irritation. The green-synthesized silver-nanoparticles-treated animals showed better wound-healing capacity from day 1 onwards and decreased wound size throughout the remaining days of treatment when compared to control group animals. At the end of the study, the wounds exhibited approximately 94% ± 1% (*p* < 0.01) constriction after treatment with C Ag NPs and 87% ± 1% (*p* < 0.01) closure in the A Ag NPs group, whereas the control wound exhibited approximately 74% ± 1% closure and the positive control (povidone–iodine) and vehicle control (Vaseline) wounds showed 79% ± 1% and 76% ± 1% wound constriction, respectively (Figure 9B and Table 2).The decreased size of wounds and increased healing efficacy could possibly be explained by the green-synthesized Ag NPs microbial efficacy towards microbial infection surrounding the region of the wound. Mondal et al. demonstrated that tissue regeneration of damaged sites is well-recognized in wound healing experiments, and the outcomes were satisfactory in earlier studies of wound constriction [59].The C Ag NPs-treated group showed an improvement in whole wound appearance following decreased irritation, as shown by alleviated inflammation and negligible bruising on day 11 of the experiment. The experimental results revealed that the healing capacity of wounds treated with *C. roseus* silver nanoparticles was greater than that of *A. indica* silver nanoparticles.

According to Tian et al., the significant action of green-synthesized Ag NPs in the healing of wounds in mice is attributed to faster regeneration, which is preferred for improved appearanceand occurs in a dose-dependent manner. In addition, green-synthesized Ag NPs showed positive results throughout the experiment due to their antibacterial efficacy and ability to decrease inflammation of wounds by diminishing the infiltration of mast cells and lymphocytes and the modification of cytokines with a fibrogenic nature [60]. Correspondingly, Liu et al. showed the efficacy of green-synthesized Ag NPs in epidermal re-epithelialization and dermal contraction, demonstrating that green-synthesized Ag NPs might enhance the percentage of wound constriction. Their characteristic wound-healing nature was explained by enhanced keratinocytes production and their movement in damaged wound sites [61]. Additionally, green-synthesized Ag NPs may possibly improve the differentiation of fibroblasts to myofibroblasts, thus increasing the healing capacity of wounds [62].

In the current study, the biosynthesized Ag NPs demonstrated significant potency in wound healing by enhancing the proliferation and migration of fibroblasts. The Ag NPs synthesized from *C. roseus* and *A. indica* enhanced the differentiation of fibroblasts into myofibroblasts and eventually improved wound contraction [63]. Nowadays, Ag NPs coated with biomedical products are commonly used to avoid microbial ailments by enhancing the healing capacity of wounds [64]. Current in vitro and in vivo studies have proven that the synthesized Ag NPs show effective antimicrobial activity against MDR bacteria that causes infections on the skin.

## 3. Materials and Methods

### 3.1. Leaf Extracts Preparation from C. roseus and A. indica

Leaf extracts from *C. roseus* and *A. indica* plants were prepared and used to synthesize silver nanoparticles. Young leaves of *C. roseus* and *A. indica* were obtained from Yogi Vemana University (14.473786° N, 78.711482° E; premises in Kadapa, Andhra Pradesh, India). Leaves were cleaned thoroughly to remove the debris and other organic constituents and dried at 37 °C. Powder was made from dried leaves, and 10 g of this powder was mixed with 100 mL distilled water (10% *w*/*v*) in a conical flask and then boiled for 1 h at 80 °C. The brown leaf extract was filtered through Whatmann No. 1 paper and preserved at 4 °C.

### 3.2. Preparation of Silver Nanoparticlesfrom Leaf Extract

Green synthesis of silver nanoparticles (AgNPs) was performed following the method of Sulaiman et al. [65]. Phytoextracts (1 mL) were mixed with different concentrations of silver nitrate (AgNO_3_) (GR Merck, India), ranging from 1–5 mM. This step was carried out at 37 °C in the dark to reduce AgNO_3_. The reduction of silver ions to silver nanoparticles was determined by the change in color to dark brown. The prepared Ag NPs were also validated using spectroscopy.

### 3.3. Microbial Strains

Prevalent MDR bacterial strains were generously gifted to us by the Microbiology Department of Yogi Vemana University. The potent drug-resistant bacterial strains of *E. coli*, *K. pneumoniae*, *S. aureus*, and *P. aeruginosa* were utilized to evaluate the antibacterial and wound healing properties of silver nanoparticles of *C. roseus* and *A. indica*.

### 3.4. Silver Nanoparticles Characterization

Ag NPs were characterized by X-ray diffraction (XRD) using Rigaku Miniflex with Cu Kα radians at 2θ angles varying from 20° to 80°. Optical properties were investigated using DRS UV Visible spectra, with a frequency varying from 500 to 4000 cm^−1^ and 4 cm^−1^ resolution.FTIR spectra were recorded by employing KBr disks using an FTIR spectrometer (Perkin Elmer, Spectrum 2) with a wave number of 4000–400 cm^−1^ [66]. Dynamic light scattering was performed using a Zetasizer-Nano ZS based on a conventional approach with several variations. Silver nanoparticles (100 μg/mol) were sonicated for 2 min, and dynamic particle sizes were assessed by adding two drops of nanoparticles into 10 mL of Millipore water. Once the nanoparticles were widely dispersed in water, the nanoparticles size was measured with a DLS analyzer. The analyses were repeated many times to attain a standard size of nanoparticles. Ag NPs (1 mg/mL) were prepared in Milli-Q water and used for further analysis. The morphology, particle size, and microstructure of Ag NPs were examined by high-resolution scanning electron microscopy [46] (Nikon, Japan). Briefly, 1 mg/mL of Ag NPs was sonicated to obtain a homogenous suspension. A sonicated stock solution was used for the size measurement of silver, which was diluted many times. Images were captured using one drop of dried gold-coated sample. Particle size, shape morphology, and fine pattern were evaluated with higher resolution TEM in a JEOL3010 (Japan) operated at 200 kV, following the protocol reported by Chattopadhyay et al. [67]. The solution was developed by adding 95% alcohol and performing 15 min of ultra-sonication. One drop of Ag NPs was placed on a carbon-coated grid and allowed to dehydrate prior to examination. TEM grids were made by adding a drop of Ag NPs onto carbon-coated Cu grids and allowing them to dry. Images were then taken.

### 3.5. Antibacterial Activity of Silver Nanoparticles

The antibacterial activities of Ag NPs were evaluated using previously isolated MDR bacterial isolates such as *E. coli*, *K. pneumoniae*, *S. aureus*, and *P. aeruginosa* from wound infection patients [12]. Different concentrations, ranging from 10 to 120 µg/µL of Ag NPs obtained from *C. roseus* and *A. indica*, were tested for antibacterial activity against MDR bacteria using the agar well diffusion method and the micro titer plate method. The positive controls used were tetracycline (30 µg), ampicillin (30 µg), and erythromycin (20 µg), and the negative control was deionized water. Antibacterial activity was measured by the zone of inhibition, and the experiment was performed in triplicate.

### 3.6. Wound Healing Activity of Silver Nanoparticles

Female BALB/c mice were procured from Mahaveera Enterprises (Reg.no:1656/PO/Bt/S/12/CPCSEA, Hyderabad, India). BALB/c mice (20–25 g) aged 8–10 weeks were housed at the YVU animal house following the laboratory animal procedures approved by the Institute Animal Ethics Committee (IAEC; CPCSEA no: 1841/GO/Re/S/51/CPCSEA). All procedures were conducted in accordance with the Guide for the Care and Use of Laboratory Animals. The anesthesia dose was 5 mL, with 2 mL ketamine (50 mg/mL), 0.5 mL xylazine (2%), and 2.5 mL saline (9%). The hair on the dorsal skin of the animalwas removed with an artificial hair removal cream [68] and wiped with 70% ethanol. Mice were anesthetized with 40–50 µL of a ketamine and xylazine mixture, depending on the weight of the animal, and a full-thickness open excision wound was made with a 5-mm biopsy punch. Following recovery from anesthesia, micewere housed separately in appropriately sanitized cages. The laboratory mice were distributed evenly and randomly separated into five groups as follows: Group I as the PBS- negative control, Group II as the betadine-positive control (povidone–iodine ointment-treated), Group III as the vehicle control (Vaseline-treated), GroupIV as 1% *w*/*w* nano-formulation-1 (*C. roseus* Ag NPs-treated), and Group V as the 1% *w*/*w* nano-formulation-2 (*A. indica* Ag NPs-treated). We pre-formulated 1 mg of green-synthesized silver nanoparticles (C Ag NPs and A Ag NPs), ground in 1 g of Vaseline (1 mg Ag NPs per 1 g Vaseline), and prepared a fine paste using a motor and pestle (~50 μL) for topical application towound surfaceson alternative days for 14 days. Wound constriction was observed by monitoring the wounds at days 0, 1, 3, 5, 7, 9, and 11, and wound closure (in mm) was measured alternatively using a digital Vernier caliper. Wound recovery efficacy is represented as the percentage of wound contraction rate (WCR).

The percentage of wound contraction rate = original wound area - actual wound area/original wound area ×100.

## 4. Conclusions

The current study demonstrates the biological production of Ag NPs via phytosynthesis using bioreductant, eco-friendly, and renewable *C. Roseus* and *A. indica* leaf extracts. Silver nanoparticleswere quickly and inexpensively synthesizedusing this method. Ag NPs were prepared from aqueous leaf extracts of *C. roseus* and *A. indica* and characterized by XRD, FT-IR, DLS, SEM-EDX, and TEM analyses and their antibacterial efficacy against MDR bacteria and ability to promote wound healing in BALB/c mice. Physical characterization methods revealed that the produced Ag NPs were extremely small and had a highly pure form in nature. Phytoderivatives such as leaf constituents and proteins of plants act as masking agents on nanoparticles. Green-synthesized Ag NPs exhibited *in-vivo* wound healing efficacy and antibacterial activity against MDR *E. coli*, *K. pneumoniae*, *S. aureus*, and *P. aeruginosa* strains. Green-synthesized Ag NPs are one alternative for the management of MDR microbial inflammation; thus, green-synthesized Ag NPs may be used for the management of wounds. Using emerging applicable nanotechnology, we synthesized metallic Ag NPs through a green route and evaluated the antibacterial and wound healing properties. Based on our current results, green-synthesized Ag NPs may have potential applications as pharmaceutical agents for antibacterial activity against MDR bacteria and wound healing.

## Figures and Tables

**Table 1:** Antibiotic susceptibility test of MDR bacteria and the zone of inhibition for different concentrations of green-synthesized silver nanoparticles.

Ag NPs Conc.	*C. roseus* Silver Nanoparticles (Mean Zone of Inhibition in mm)				*A. indica* Silver Nanoparticles (Mean Zone of Inhibition in mm)			
	EC	KP	SA	PA	EC	KP	SA	PA
10 μg/μL	7	11	8	6	4	4	2	2
30 μg/μL	11	13	12	12	6	5	5	5
60 μg/μL	12	16	15	14	9	11	9	8
90 μg/μL	15	17	15	16	10	15	12	10
120 μg/μL	17	19	19	17	12	16	14	12

EC—*Escherichia coli*, KP—*Klebsiella pneumoniae*, SA—*Staphylococcus aureus*, and PA—*Pseudomonas aeruginosa*.

**Table 2:** Percentage of wound contraction in BALB/c mice excision wound model. Note: mean± SE.

Group	Treatment Groups	% of Wound Contraction in Days					
		1	3	5	7	9	11
Group-I	Control	4 ± 1	24 ± 4	40 ± 5	48 ± 3	65 ± 3	74 ± 1
Group-II	Betadine (Povidone–Iodine)	5 ± 1	23 ± 3	32 ± 5	53 ± 4	69 ± 1	79 ± 1
Group-III	Vaseline	4 ± 1	24 ± 3	36 ± 3	55 ± 2	68 ± 1	76 ± 1
Group-IV	1% *C. roseus* Ag NPs	5 ± 1	29 ± 3	45 ± 3	60 ± 2	83 ± 1	94 ± 1
Group-V	1% *A. indica* Ag NPs	5 ± 1	23 ± 2	46 ± 2	59 ± 4	78 ± 2	87 ± 1
